# Large Language Model Simplification of Open Access Pediatric Strabismus Literature: Cross-Sectional Validation of Readability and Clinical Fidelity

**DOI:** 10.2196/91572

**Published:** 2026-08-14

**Authors:** Mingming Jiang, Mingming Zhou, Xiaomei Wan, Jing Zhang

**Affiliations:** 1 Eye Institute of Shandong First Medical University, Qingdao Eye Hospital of Shandong First Medical University, No.5 Yan'erdao Road, Shinan District, Qingdao, Shandong, 266000, China, 86 15898825081; 2 State Key Laboratory Cultivation Base, Shandong Key Laboratory of Eye Diseases, Qingdao, Shandong, China; 3 School of Ophthalmology, Shandong First Medical University, Qingdao, Shandong, China

**Keywords:** large language model, LLM, pediatric strabismus, Flesch-Kincaid Grade Level, FKGL, Simple Measure of Gobbledygook, SMOG, readability, clinical fidelity

## Abstract

**Background:**

Peer-reviewed medical literature consistently violates established health literacy readability targets, creating a gap that effectively excludes patients and caregivers from accessing evidence-based information.

**Objective:**

This study aimed to evaluate whether a large language model (LLM) can generate plain-language summaries of pediatric strabismus literature while preserving clinical fidelity and meeting established health literacy readability targets.

**Methods:**

This cross-sectional study analyzed 85 open access, peer-reviewed pediatric strabismus articles published between 2022 and 2025, stratified by strabismus subtype, surgical relevance, and publication type. Full-text articles were processed using DeepSeek-V3 (DeepSeek) via a structured prompt, which instructed the model to provide a simplified summary meeting the following requirements for each article: a seventh-grade or lower reading level, a maximum length of 800 words, and strict preservation of medically significant data. Primary outcomes were readability scores measured by the Flesch-Kincaid Grade Level (FKGL) and Simple Measure of Gobbledygook (SMOG) indices. Secondary outcomes included clinical fidelity, which was independently assessed by 2 fellowship-trained pediatric strabismus specialists.

**Results:**

Baseline articles demonstrated a mean FKGL score of 15.79 (SD 1.53) and a mean SMOG score of 14.41 (SD 1.09). Following LLM simplification, the mean FKGL score significantly decreased from 15.79 (SD 1.53) to 7.84 (SD 1.30), representing a mean difference of 7.95 (95% CI 7.52-8.38; *P*<.001). Similarly, the mean SMOG score decreased from 14.41 (SD 1.09) to 7.68 (SD 0.94), representing a mean difference of 6.73 (95% CI 6.42-7.04; *P*<.001). Postsimplification readability did not differ significantly by strabismus subtype or surgical relevance (all adjusted *P*>.05). However, case reports retained slightly higher FKGL scores (mean 8.35, SD 0.89) compared to reviews (mean 7.89, SD 1.37) and original research (mean 7.48, SD 1.40) (adjusted *P*=.003). Out of the 85 summaries, clinical fidelity was rated good in 81 (95.29%), moderate in 4 (4.71%; these were exclusively summaries of review articles), and poor in 0 (0%).

**Conclusions:**

DeepSeek-V3 effectively reduced the reading level of complex pediatric strabismus literature by approximately 8 grade levels, achieving National Institutes of Health–recommended eighth-grade or lower targets without compromising clinical accuracy. When integrated with clinician oversight, LLM-generated summaries offer a scalable, equitable tool to enhance health literacy and support shared decision-making for patients and caregivers.

## Introduction

Health literacy fundamentally influences patient decision-making and health outcomes [1]. The American Medical Association (AMA) recommends a sixth-grade reading level for patient educational materials, while the National Institutes of Health (NIH) recommends an eighth-grade or lower readability level to ensure broad comprehension [2,3]. However, peer-reviewed medical literature consistently violates this standard, with publications averaging 14th- to 16th-grade reading levels [4-7]. This readability gap effectively excludes patients from accessing evidence-based information that could inform their care decisions.

Within ophthalmology, strabismus represents a domain with particularly complex terminology and conceptual frameworks. Unlike common ocular conditions, strabismus encompasses specialized concepts, such as dissociated vertical deviation, the accommodative convergence/accommodation ratio, and monofixation syndrome [8,9]. These linguistic barriers persist even among health care professionals outside pediatric ophthalmology. Given that strabismus affects 2% to 4% of children globally and requires timely intervention during critical developmental periods [9], this communication challenge significantly impedes shared decision-making and treatment adherence.

Recent advances in AI, particularly in large language models (LLMs) such as GPT-4, demonstrate promising capabilities in translating complex medical content into accessible language while maintaining clinical accuracy across diverse specialties, including oncology [10], rheumatology [11], pediatric emergency medicine [12], and shoulder-elbow surgery [13]. In ophthalmology, preliminary studies have validated this approach across various subspecialties. LLMs have been deployed to create patient educational materials for uveitis [14], strengthen patient and caregiver education in pediatric ophthalmology [15-20], and improve understanding of peer-reviewed literature—lowering readability by roughly 8 grade levels without introducing factual inaccuracies [21]. However, no studies to date have specifically investigated LLM applications in strabismus-related literature, a field where specialized terminology and time-sensitive developmental concerns amplify the impacts of limited health literacy. This study presents the first systematic evaluation of whether LLMs can effectively bridge the readability gap in literature on pediatric strabismus while preserving clinical fidelity, where fidelity is defined as the consistency of LLM-generated content with objective facts, user instructions, and source information.

Accordingly, this study aims to (1) evaluate whether the open-source LLM DeepSeek-V3 (DeepSeek) can simplify open access pediatric strabismus literature to eighth-grade or lower readability while preserving all medically significant data, (2) quantify the magnitude of readability improvement across different strabismus subtypes, surgical topics, and article types, and (3) assess clinical fidelity through independent expert review. We hypothesized that LLM-simplified summaries would achieve near-target readability (eighth grade or lower) with high fidelity (≥90% rated good) but that case reports and review articles might present differential challenges due to their inherent linguistic complexity.

## Methods

### Ethical Considerations

This cross-sectional study involved only the secondary analysis of publicly available, deidentified, open access, peer-reviewed literature. As this study did not involve any human participants, animal subjects, or primary data collection, it does not meet the definition of human subjects research. In accordance with Article 32 of Measures for the Ethical Review of Life Science and Medical Research Involving Humans [22], which stipulates that secondary analysis of publicly published literature is exempt from ethical review, this study was exempt from institutional review board approval and informed consent requirements. The study was conducted in compliance with the ethical principles outlined in the Declaration of Helsinki for scholarly research.

### Article Selection and Inclusion Criteria

This study was conducted and reported in accordance with the PRISMA (Preferred Reporting Items for Systematic Reviews and Meta-Analyses) 2020 guidelines for transparent literature identification [23]. A comprehensive search of the PubMed database was executed on October 15, 2025, to identify English-language, open access articles published between January 1, 2022, and September 30, 2025. The search strategy combined MeSH and free-text terms: (“strabismus” [title/abstract] OR “esotropia” [title/abstract] OR “exotropia” [title/abstract] OR “intermittent exotropia” [title/abstract] OR “infantile esotropia” [title/abstract] OR “strabismus” [MeSH terms]) AND (“child*”[title/abstract] OR “pediatric*” [title/abstract] OR “child” [MeSH terms] OR “adolescent” [MeSH terms]).

Eligible articles were required to meet all of the following criteria: (1) study design, (2) accessibility, and (3) text length. First, articles had to report original research (including prospective/retrospective cohort studies, case-control studies, and randomized trials), case reports, or narrative or systematic reviews. Second, articles had to be freely available under a Creative Commons license or publisher open access policy. Third, articles had to include at least 300 words of continuous narrative text in the main body (title, abstract, introduction, methods, results, and discussion); this word count excluded references, tables, figure legends, and supplementary materials. Exclusion criteria comprised non–open access articles and articles that comprised more than 50% non-English quotations or untranslated foreign-language excerpts.

Search results were exported to EndNote (version 20; Clarivate Plc) for automated deduplication followed by manual verification. Two independent investigators (MZ and MJ) screened titles and abstracts against predefined eligibility criteria. Discrepancies were resolved through consensus discussion or, when necessary, consultation with a third investigator (JZ). Full-text articles of potentially eligible records were retrieved and assessed in duplicate.

### Simplification Protocol

The simplification task was performed using DeepSeek-V3. For each article, the full main text (title, introduction, methods, results, and discussion) was submitted with the following structured prompt:

Please rephrase the content of the peer-reviewed scientific article I provide to ensure comprehension by readers at a middle school education level. The adapted text should incorporate the following elements: Preserve all medically significant numerical data; Convert specialized terminology into everyday language; Maintain factual accuracy without introducing external information; Adhere to a maximum length of 800 words.

Each article was processed in a dedicated, isolated inference session (ie, no cross-article context retention), and only the first generated output was retained for analysis.

### Readability Assessment

Readability was quantified using 2 validated, widely adopted indices in health communication research: the Flesch-Kincaid Grade Level (FKGL) and the Simple Measure of Gobbledygook (SMOG). Assessments were performed using an online readability calculator [24].

Two fellowship-trained pediatric strabismus specialists, provided with both the original texts and the corresponding LLM-generated summaries, independently assessed the clinical fidelity of each LLM-simplified summary by comparing it against the original source text, categorizing it as good (all key concepts, data, and conclusions preserved without distortion or addition), moderate (most content retained, minor omissions, no substantive misrepresentation), or poor (critical omissions, misinterpretation of meaning, or unsupported additions).

### Statistical Analysis

All statistical analyses were conducted using R software (version 4.3.1; R Foundation for Statistical Computing). The primary prespecified hypothesis tested the overall reduction in readability scores (FKGL and SMOG) following LLM processing. Continuous variables are reported as mean (SD) with 95% CIs for mean differences. Pre- vs post-LLM comparisons were performed using paired 2-tailed *t* tests.

All additional comparisons across strabismus subtypes, surgical relevance, and article types were explicitly designated as exploratory and were conducted to characterize potential heterogeneity in model performance rather than to test prespecified hypotheses. To address the multiple-comparisons problem inherent in these exploratory analyses and strictly control the type I error rate, *P* values for all subgroup tests were adjusted using the Benjamini-Hochberg false discovery rate (FDR) procedure; adjusted *P*<.05 was considered statistically significant. Given the exploratory nature of these comparisons and the reduced sample sizes following stratification, subgroup findings should be interpreted as hypothesis generating rather than confirmatory. Primary findings remain robust due to large effect sizes (ΔFKGL≈7.95 grade levels). Fidelity ratings were analyzed descriptively owing to near-perfect interrater agreement (Cohen κ was not calculable due to negligible discordance). All statistical tests were 2-sided, and α=.05. Exact *P* values are reported to 2 decimal places unless <.01.

## Results

### Baseline Literature Characteristics

**Figure 1.**
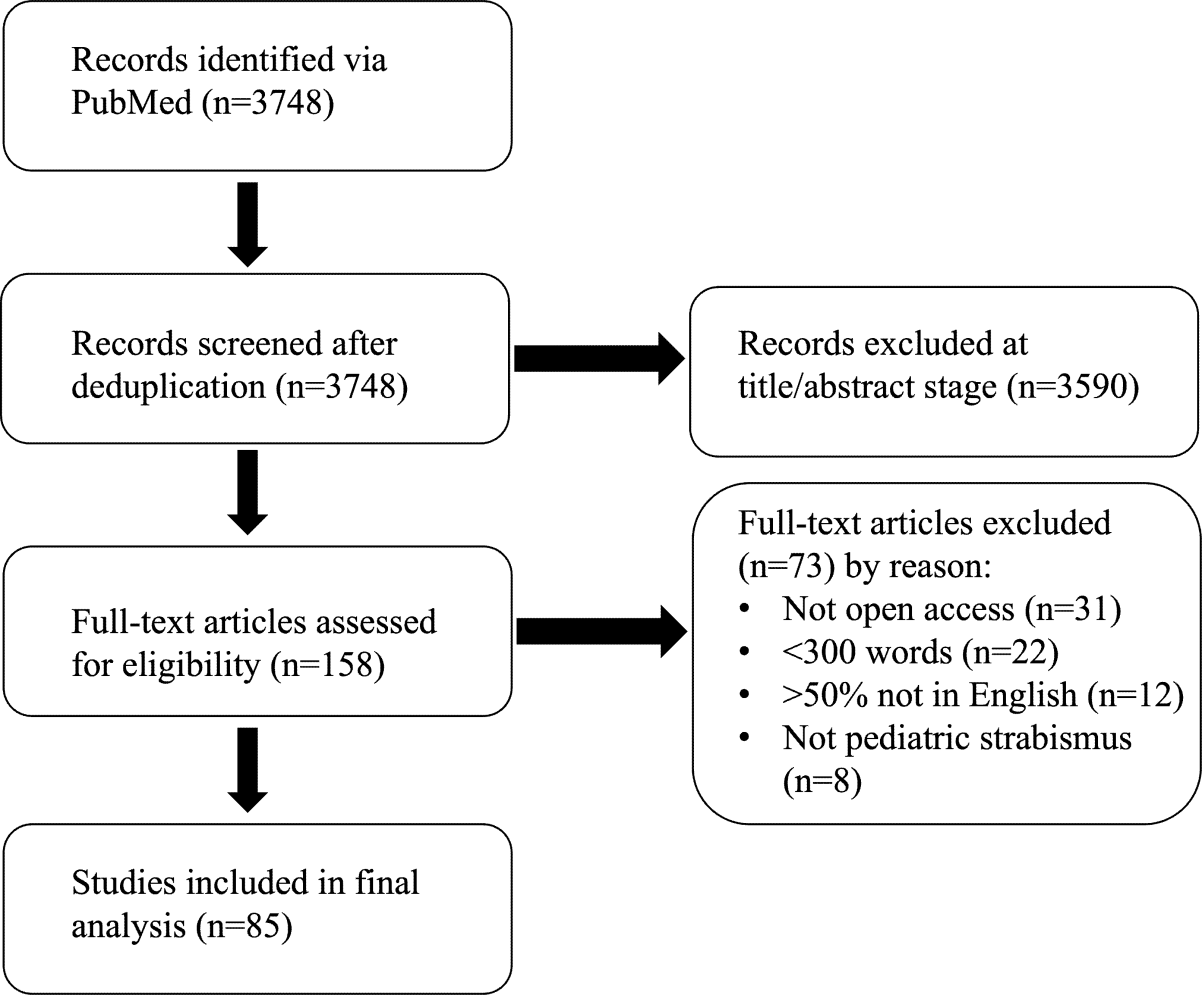
PRISMA (Preferred Reporting Items for Systematic Reviews and Meta-Analyses) 2020 flow diagram of the literature search and study selection process.

A total of 85 open access, peer-reviewed articles on pediatric strabismus published between 2022 and 2025 were included (the complete screening workflow, including exclusion reasons at each stage, is summarized in Figure 1). Strabismus subtypes comprised esotropia (n=25, 29.4%), exotropia (n=31, 36.5%), and other types (n=29, 34.1%). Surgical topics appeared in 45 articles (52.9%) and nonsurgical topics in 40 articles (47.1%). Article types included case reports (n=26, 30.6%), reviews (n=19, 22.4%), and original research (n=40, 47.1%). The mean FKGL score was 15.79 (SD 1.53), and the mean SMOG score was 14.41 (SD 1.09). No statistically significant differences in mean FKGL or SMOG scores were observed across strabismus subtypes, surgical relevance, or article type (all *P*>.05; Table 1).

**Table 1.** The basic characteristics of the included literature. *P* values were calculated using 1-way ANOVA or independent *t* tests for continuous variables across subgroups.

	Articles, n (%)	FKGL,^a^ mean (SD)	SMOG,^b^ mean (SD)
Total	85 (100)	15.79 (1.53)	14.41 (1.09)
Publication year			
2022‐2023	33 (38.82)	—^c^	—
2024‐2025	52 (61.18)	—	—
Strabismus type			
Esotropia	25 (29.41)	15.92 (1.82)	14.52 (1.39)
Exotropia	31 (36.47)	15.68 (1.19)	14.35 (0.91)
Other	29 (34.12)	15.79 (1.61)	14.38 (1.01)
*P* value	—	.84	.84
Surgery			
Surgery related	45 (52.94)	15.58 (1.44)	14.42 (1.03)
Non–surgery related	40 (47.06)	16.03 (1.61)	14.40 (1.17)
*P* value	—	.18	.93
Article type			
Case reports	26 (30.59)	15.62 (1.65)	14.42 (1.06)
Reviews	19 (22.35)	16.16 (1.61)	14.53 (1.17)
Original research	40 (47.06)	15.73 (1.41)	14.35 (1.10)
*P* value	—	.47	.85

a FKGL: Flesch-Kincaid Grade Level.

b SMOG: Simplified Measure of Gobbledygook.

c Not applicable.

### Readability After LLM Processing

**Figure 2.**
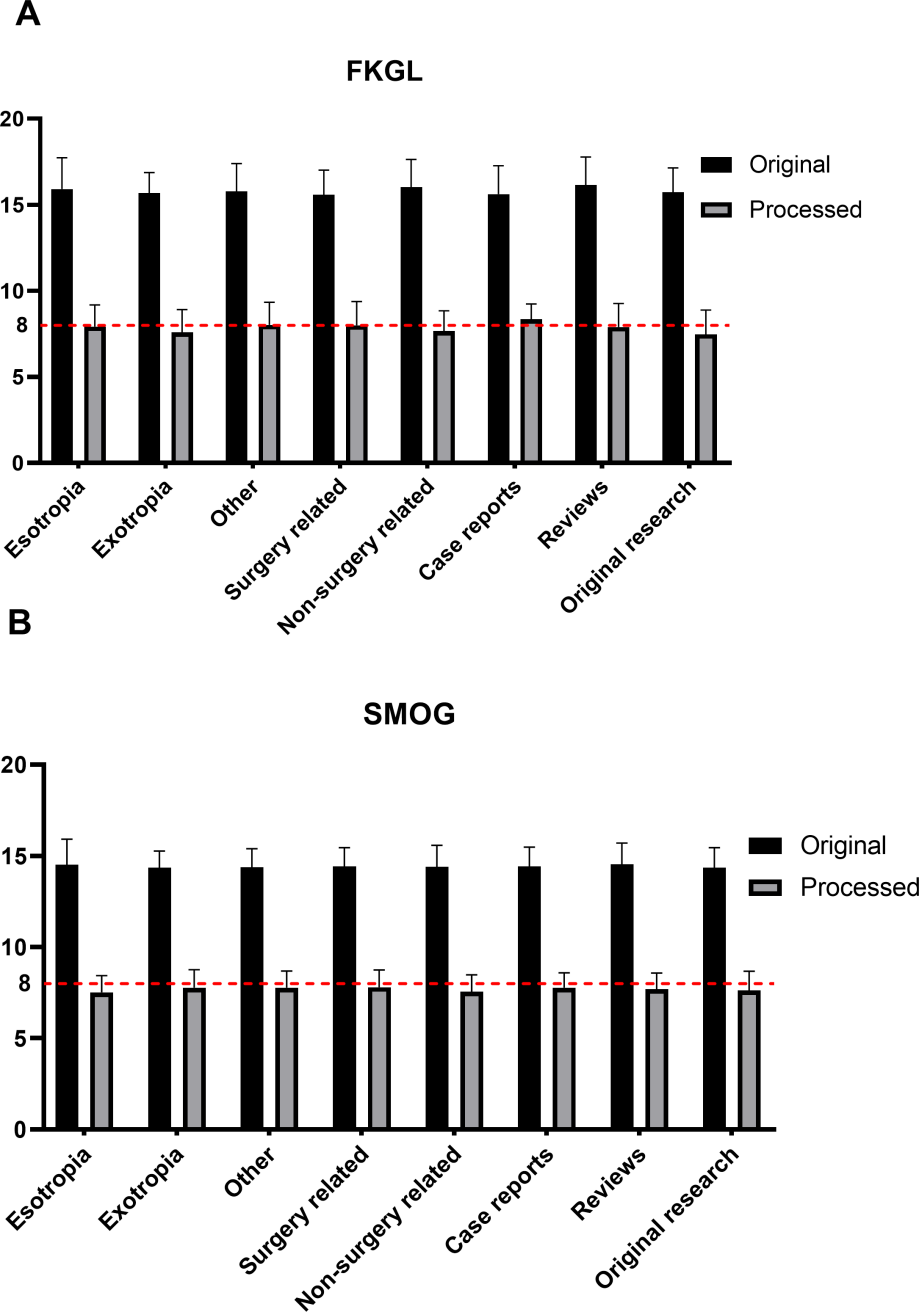
Readability comparison before and after large language model (LLM) simplification: (A) Flesch-Kincaid Grade Level (FKGL) scores for original and LLM-processed articles across article types. (B) Simple Measure of Gobbledygook (SMOG) scores for original and LLM-processed articles across article types. Dark bars: original articles; light bars: LLM-processed summaries. Red dashed horizontal line indicates National Institutes of Health–recommended eighth-grade or lower readability threshold.

Following LLM processing, the mean FKGL score decreased significantly by 7.95 grade levels (95% CI 7.52-8.38; *P*<.001), and the mean SMOG score decreased by 6.73 grade levels (95% CI 6.42-7.04; *P*<.001). In exploratory subgroup analyses (adjusted for multiple comparisons using the Benjamini-Hochberg FDR procedure), FKGL values for articles postprocessing remained statistically indistinguishable across strabismus subtypes (esotropia: 7.92, SD 1.26; exotropia: 7.58, SD 1.34; other: 8.03, SD 1.30; adjusted *P*>.05) and across surgical relevance (surgery related: 8.00, SD 1.38; non–surgery related: 7.65, SD 1.19; adjusted *P*>.05). However, a significant between-group difference was observed by article type (case reports: 8.35, SD 0.89; reviews: 7.89, SD 1.37; original research: 7.48, SD 1.40; adjusted *P*=.003). Post hoc inspection indicated that case reports retained slightly higher FKGL scores after simplification, though all subgroup means remained within the NIH-recommended eighth-grade or lower threshold. SMOG values showed no significant between-group differences across any stratification (all adjusted *P*>.05). Details are shown in Table 2 and Figure 2.

**Table 2.** Readability analysis of articles before and after large language model (LLM) processing.

	Original, mean (SD)	Processed, mean (SD)	Mean difference^a^ (95% CI)	*P* value^b^
Overall analysis				
FKGL^c^	15.79 (1.53)	7.84 (1.30)	7.95 (7.52-8.38)	<.001
SMOG^d^	14.41 (1.09)	7.68 (0.94)	6.73 (6.42-7.04)	<.001
FKGL subgroup analysis^e^				
Strabismus type				.56
Esotropia	15.92 (1.82)	7.92 (1.26)	8.00 (7.11-8.90)	<.001
Exotropia	15.68 (1.19)	7.58 (1.34)	8.10 (7.45-8.74)	<.001
Other	15.79 (1.61)	8.03 (1.30)	7.76 (6.99-8.53)	<.001
Surgery				.45
Surgery related	15.58(1.44)	8.00 (1.38)	7.58 (6.99-8.17)	<.001
Non–surgery related	16.03 (1.61)	7.65 (1.19)	8.38 (7.75-9.01)	<.001
Article type				.003
Case reports	15.62 (1.65)	*8.35 (0.89)* ^f^	7.27 (6.53-8.01)	<.001
Reviews	16.16 (1.61)	*7.89 (1.37)*	8.26 (7.28-9.25)	<.001
Original research	15.73 (1.41)	*7.48 (1.40)*	8.25 (7.63-8.88)	<.001
SMOG subgroup analysis^e^				
Strabismus type				.70
Esotropia	14.52 (1.39)	7.52 (0.92)	7.00 (6.33-7.67)	<.001
Exotropia	14.35 (0.91)	7.77 (0.99)	6.58 (6.10-7.07)	<.001
Other	14.38 (1.01)	7.77 (0.92)	6.66 (6.15-7.17)	<.001
Surgery				.45
Surgery related	14.42 (1.03)	7.80 (0.94)	6.63 (6.21-7.04)	<.001
Non–surgery related	14.40 (1.17)	7.55 (0.93)	6.85 (6.38-7.32)	<.001
Article type				.83
Case reports	14.42 (1.06)	7.77 (0.82)	6.65 (6.13-7.18)	<.001
Reviews	14.53 (1.17)	7.68 (0.89)	6.84 (6.16-7.53)	<.001
Original research	14.35 (1.10)	7.63 (1.05)	6.73 (6.25-7.20)	<.001

a 95% CI for the mean difference between pre- and postprocessing scores. Mean differences were calculated using unrounded raw data, which may result in minor discrepancies when subtracting the rounded means shown in the table.

b *P* values adjusted for multiple comparisons using Benjamini-Hochberg false discovery rate (FDR) procedure; adjusted *P*<.05 considered significant.

c FKGL: Flesch-Kincaid Grade Level.

d SMOG: Simple Measure of Gobbledygook.

e Subgroup analyses are exploratory.

f Italics indicate statistically significant between-group difference after FDR correction.

### Word Count Distribution

Original articles averaged 2786.84 (SD 673.99) words; LLM-processed outputs averaged 724.28 (SD 149.65) words (*P*<.001). By article type, word counts were as follows: preprocessed case reports, mean 1444.42 (SD 599.17) vs postprocessed case reports, mean 827.81 (SD 121.75; *P*<.001); preprocessed reviews, mean 4206.28 (SD 3800.29) vs postprocessed reviews, mean 716.94 (SD 165.70; *P*<.001); and preprocessed original research, mean 2826.55 (SD 743.66) vs postprocessed original research, mean 659.08 (SD 123.10; *P*<.001). Between-group comparison of original word counts revealed significant heterogeneity (*P*<.001, 1-way ANOVA), which persisted after LLM processing (adjusted *P*=.006; Table 3).

**Table 3.** Word count analysis before and after large language model (LLM) processing.

	Original, mean (SD)	Processed, mean (SD)	*P* value^a^
All articles	2786.84 (1673.99)	724.28 (149.65)	<.001
Case reports	1444.42 (599.17)	827.81 (121.75)	<.001
Reviews	4206.28 (3800.29)	716.94 (165.70)	<.001
Original research	2826.55 (743.66)	659.08 (123.10)	<.001
*P* value	<.001^b,c^	.006^c^	—^d^

a Paired *t* test within each subgroup.

b One-way ANOVA comparing original word counts across article types.

c Adjusted *P* values for processed word counts via Benjamini-Hochberg false discovery rate (FDR).

d Not applicable.

### Fidelity Assessment

In terms of fidelity, 81 of 85 summaries (95.3%) were rated good, 4 (4.7%) were rated moderate, and none were rated poor. All case reports (n=26) and original research articles (n=40) achieved good ratings. Among reviews, 15 (78.9%) were rated good and 4 (21.1%) were rated moderate. Fidelity did not differ significantly by surgical status or strabismus subtype. Interrater agreement was near perfect, with all initial discrepancies resolved through consensus discussion (Cohen κ was not calculable due to negligible discordance; Table 4).

**Table 4.** Fidelity ratings of large language model (LLM)–simplified summaries.

	Good,^a^ n (%)	Moderate,^b^ n (%)	Poor,^c^ n (%)
Overall analysis (n=85)	81 (95.29)	4 (4.71)	0 (0)
Subgroup analysis			
Article type			
Case reports (n=26)	26 (100)	0 (0)	0 (0)
Reviews (n=19)	15 (78.95)	4 (21.05)	0 (0)
Original research (n=40)	40 (100)	0 (0)	0 (0)
Surgery			
Surgery related (n=45)	43 (95.56)	2 (4.44)	0 (0)
Non–surgery related (n=40)	38 (95)	2 (5)	0 (0)
Strabismus type			
Esotropia (n=25)	23 (92)	2 (8)	0 (0)
Exotropia (n=31)	31 (100)	0 (0)	0 (0)
Other (n=29)	27 (93.10)	2 (6.90)	0 (0)

a Good: all key concepts and data preserved without distortion.

b Moderate: minor omissions, no substantive misrepresentation.

c Poor: critical omissions or misinterpretation.

## Discussion

The LLM we used (DeepSeek-V3) demonstrated considerable capacity to enhance the readability of pediatric strabismus literature. In our analysis, the mean FKGL decreased from 15.79 to 7.84, while the SMOG index declined from 14.41 to 7.68, reflecting an improvement equivalent to approximately 8 US grade levels. This magnitude of readability enhancement aligns with prior studies using LLMs in other medical domains [10-21].

Notably, whereas earlier investigations predominantly used proprietary models such as ChatGPT (OpenAI), our study deliberately selected DeepSeek. This decision was driven by our objective to serve a diverse population of patients and caregivers across varying socioeconomic strata. DeepSeek is open source and freely accessible, thereby eliminating the financial barriers inherent to commercial models. Such accessibility supports equitable dissemination of high-quality health information regardless of users’ economic resources. Moreover, emerging evidence indicates that DeepSeek exhibits robust performance in medical applications [25-28], with information processing and reasoning capabilities comparable to those of other LLMs [29,30].

DeepSeek exhibited differential performance across article types. In our study, case reports (8.35, SD 0.89) showed higher postsimplification FKGL scores, indicating poorer readability, whereas Kianian et al [21] reported no such variation, possibly because their corpus encompassed broader ophthalmic subspecialties. Notably, case reports—despite their shorter original length—yielded the longest simplified outputs (827.81, SD 121.75 words) and the highest FKGL scores. This slight exceedance of the 800-word limit specified in our prompt can be attributed to an inherent tension between 2 core instructions: “simplify to a middle-school reading level” and “strictly preserve all medically significant data.” Case reports are characterized by a high density of patient-specific quantitative data. When forced to simplify complex terminology while retaining every critical numerical value, the model inevitably requires additional explanatory phrasing to maintain clarity. Thus, the model implicitly prioritized clinical fidelity over strict length adherence. This trade-off is clinically justifiable, as it perfectly aligns with our finding that 100% (26/26) of the simplified case reports achieved a good fidelity rating, ensuring that no critical diagnostic or therapeutic information was lost in the simplification process.

Fidelity is central to model credibility and utility. In our study, 2 board-certified ophthalmologists assessed the fidelity of model-generated summaries. They found that 95.3% demonstrated good fidelity, lower than other specialties [21,31]. All 4 summaries rated as having moderate fidelity were of systematic or narrative reviews. This likely reflects the challenge of condensing comprehensive reviews (mean original length 4206 words) while preserving nuanced perspectives and multiple viewpoints. The tension between brevity and comprehensiveness is especially pronounced in review articles, potentially accounting for minor omissions despite overall accuracy.

This study has several limitations. First, only open access articles were included, potentially omitting high-impact, subscription-only publications that may offer deeper clinical insights—reflecting practical and copyright-related constraints on text use. Second, although ophthalmologists validated the accuracy and appropriateness of the simplified materials, we did not evaluate comprehension or usability among the target audience: parents of children with strabismus and adolescent patients. Additionally, readability assessments may differ from the actual perceived understandability of patients or caregivers [32,33].

This study was designed as a necessary first step to establish proof-of-concept and clinical fidelity benchmarks within pediatric strabismus literature. Future research should directly compare multiple LLM architectures (eg, DeepSeek, GPT-4, Llama) to identify optimal models for this domain, evaluate LLM-generated summaries against human-written plain-language summaries in randomized noninferiority designs, assess comprehension and usability among parents of children with strabismus and adolescent patients with strabismus, and develop patient-facing tools that support on-demand LLM simplification with integrated clinician oversight.

In conclusion, LLMs show promise for converting complex strabismus literature—especially original research and non–surgery-related studies—into patient-oriented educational materials. Although the postprocessed reading level (approximately seventh to eighth grade) is slightly above the AMA’s recommended sixth-grade level, it represents a substantial simplification from the original text and remains within the NIH’s acceptable upper limit for patient materials. With expert clinical review, these summaries may support shared decision-making by caregivers and adolescent patients. Pending direct validation with target users, clinician oversight remains necessary before deploying LLM-generated content in clinical practice.

## Abbreviations

AMA: American Medical Association
FDR: false discovery rate
FKGL: Flesch-Kincaid Grade Level
GAIDeT: Generative Artificial Intelligence Delegation Taxonomy
LLM: large language model
NIH: National Institutes of Health
PRISMA: Preferred Reporting Items for Systematic Reviews and Meta-Analyses
SMOG: Simple Measure of Gobbledygook
